# Functional acuity score as a surrogate of reading performance in low vision: A Japanese disability evaluation cohort study

**DOI:** 10.1371/journal.pone.0358323

**Published:** 2026-09-11

**Authors:** Mieko Tsuruoka, Kenji Inoue

**Affiliations:** Inouye Eye Hospital, Tokyo, Japan; Marshall B. Ketchum University, UNITED STATES OF AMERICA

## Abstract

This retrospective cross-sectional study examined whether the Functional Acuity Score (FAS), an administrative impairment measure calculated from routine visual acuity data, was associated with reading performance in individuals undergoing formal visual disability evaluation. Thirty-one participants applying for visual disability certification at a tertiary eye hospital in Japan underwent Goldmann perimetry and MNREAD-J testing within 12 months. FAS, the Functional Field Score (FFS), and the Functional Vision Score (FVS) were calculated according to the AMA Guides. Their associations with reading outcomes, including critical print size (CPS) and the reading accessibility index (ACC), were assessed using correlation and multivariable regression analyses. FAS was strongly correlated with better-eye visual acuity (rho = 0.963) and showed the strongest associations with reading performance among the impairment measures. It was associated with CPS (rho = −0.82) and ACC (rho = 0.69) and remained associated with both outcomes in adjusted regression models, with R^2^ values of up to 0.76 after FAS was included. Field-based measures showed weak or inconsistent associations with reading parameters. The cohort consisted mainly of patients with glaucoma and other non-macular conditions (27/31, 87.1%). In this predominantly non-macular disability-evaluation cohort, FAS may provide an estimate of resolution-dependent reading performance when direct assessment is not available. However, only 4 of 31 participants had macular disease, and whether these findings apply to patients with macular pathology remains unclear.

## Introduction

Assessing visual disability requires measures that link organ-level function to performance in everyday activities. The World Health Organization’s International Classification of Functioning, Disability and Health (ICF) distinguishes between impairments in body functions and limitations in activities [1]. In ophthalmology, visual acuity and visual field tests capture organ-level function, whereas patients usually seek care because of difficulties with tasks such as reading, mobility, and face recognition [2]. Among these, reading is one of the most commonly reported problems and is closely tied to independence and quality of life [3].

To address the gap between clinical measures and daily functioning, composite indices have been developed that combine acuity and visual field information into standardized scores. The Functional Acuity Score (FAS) and Functional Field Score (FFS), which together form the Functional Vision Score (FVS), are calculated from routine clinical data and are used in disability certification in several countries, including Japan [4]. Because FAS is based on visual acuity, which is closely related to spatial resolution, it may be associated with resolution-dependent aspects of reading performance. However, it remains unclear whether routinely collected administrative measures provide information about activity-level reading performance beyond that obtained from dedicated reading assessments. This question has not been systematically examined.

Reading assessment is central to low-vision care. The MNREAD acuity charts provide clinically relevant measures, including critical print size (CPS) and the reading accessibility index (ACC) [5–9], both of which inform magnification strategies. Although digital tools have improved efficiency [10], reading tests are not always feasible in routine clinical practice. In addition, visual function is often measured monocularly, whereas reading is typically performed binocularly; in low vision, binocular performance does not always align with monocular acuity [11]. As a result, FAS and similar scores already derived for disability evaluation may offer some information about reading performance when direct assessment is not available.

The primary aim of this study was to examine whether FAS, an administrative measure used in formal visual disability evaluations in Japan, was associated with resolution-dependent reading performance.

## Materials and methods

### Study design and participants

This retrospective cross-sectional study reviewed the medical records of patients who applied for a physical disability certificate due to visual impairment at Inouye Eye Hospital (Tokyo, Japan) between January 2019 and March 2024. Applicants for certification represent individuals with clinically stable and functionally significant vision loss under Japanese administrative criteria; therefore, this cohort reflects patients undergoing formal disability evaluation rather than an unselected low-vision clinic population.

Participants were eligible if they had completed both Goldmann perimetry (GP) and MNREAD-J testing within a 12-month interval. This interval was permitted in accordance with the Japanese requirement for “symptom fixation,” which requires at least one year of clinical stability before permanent disability certification is granted [12]. Only patients considered clinically stable at the time of certification were included. Individuals younger than 18 years were excluded.

The study adhered to the tenets of the Declaration of Helsinki and was approved by the Institutional Review Board of Inouye Eye Hospital (Approval No. 202501−4). Given the retrospective design, the requirement for written informed consent was waived. Study information was made available on the institutional website, and participants were given the opportunity to opt out. The data were accessed for research purposes between February 2025 and September 2025. During this period, the authors had access to information that could identify individual participants; however, all data were anonymized prior to analysis.

### Visual function assessment

Age, sex, and primary ocular diagnosis were extracted from the medical records. Diagnoses were grouped into glaucoma, macular disease, optic neuropathy, and other conditions for analysis.

The Functional Vision Score (FVS) and its components—the Functional Acuity Score (FAS) and Functional Field Score (FFS)—were calculated according to the American Medical Association Guides to the Evaluation of Permanent Impairment (6th Edition) [4]. All scores were derived as weighted binocular values following the AMA protocol.

### Functional acuity score (FAS)

Distance visual acuity was measured with Landolt C optotypes using a retroilluminated visual acuity chart built into the examination unit (CV-5000; Tomey Corporation, Nagoya, Japan). Ambient illuminance during the measurement was 220 lux, as measured with a lux meter. Acuity values were converted to logMAR and then to FAS using the conversion tables provided in the AMA Guides. The scale ranges from 0 (blindness) to 100 (normal vision).

Visual acuity in decimal notation (VA) was converted to a Visual Acuity Score (VAS) using the following formula: $VAS=100+50\times \log_{10}(VA)$, which is mathematically equivalent to VAS = 100 − 50 × logMAR.

A continuous VAS value was calculated for each visual acuity measurement using this formula and was then used to calculate the Functional Acuity Score (FAS) using the formula described below [4].

Monocular logMAR values were first converted to Visual Acuity Scores (VAS). Because binocular visual acuity (VAS_OU) is not routinely measured in Japanese clinical practice, the better-eye VAS was used as a proxy for VAS_OU in all participants, consistent with standard procedures in disability certification. FAS was then calculated as a weighted combination of these values using the following formula:


$$\begin{array}{c} FAS=(3\times VAS\_OU+VAS\_OD+VAS\_OS)/5 \end{array}$$

Under this approach, FAS primarily reflects weighted monocular acuity rather than true binocular acuity.

### Functional field score (FFS)

Visual fields were assessed using Goldmann perimetry with the III4e isopter. FFS was calculated in accordance with the AMA Guides (6th Edition) [4]. The reproducibility of this method has been reported previously, with excellent intra- and interrater reliability (intraclass correlation coefficient > 0.98) using the same III4e protocol [13].

To separate overall field extent from the acuity-related adjustment specified in the AMA Guides, two field measures were defined:

- Raw FFS: calculated from isopter extent without applying the central scotoma rule
- Adjusted FFS: calculated with the central scotoma rule applied, incorporating the effect of central vision loss into the field score [4]

The final FVS was calculated using the adjusted FFS, consistent with the standard AMA method.

### Comparison with Japanese disability certification criteria

The Japanese visual disability certification system uses a similar weighting approach for visual field assessment. Under the revised 2016 criteria issued by the Ministry of Health, Labour and Welfare, the binocular central visual field angle is calculated as [12]:


$$\begin{array}{c} \text{Binocular\textasciitilde{}central\textasciitilde{}visual\textasciitilde{}field\textasciitilde{}angle}=(3\times \text{larger-eye\textasciitilde{}angle}+\text{smaller-eye\textasciitilde{}angle})/4 \end{array}$$

This formulation parallels the AMA approach to the Functional Field Score (FFS), in which greater weight is assigned to the better-performing eye.

The Japanese guidelines also state that binocular visual acuity is not routinely measured in standard ophthalmic practice; instead, better-eye visual acuity is used for disability grading. This practice supports the approach adopted in the present study, where better-eye Visual Acuity Score (VAS) was used as a proxy for binocular VAS in calculating the Functional Acuity Score (FAS).

Unlike the AMA framework, however, the Japanese system does not combine acuity and visual field into a single composite measure equivalent to the Functional Vision Score (FVS). Visual acuity and visual field are evaluated separately, and the overall disability grade is determined by the more severe impairment, with upward adjustment when both criteria are met.

### Reading performance assessment

Reading performance was assessed using the MNREAD-J application [7,10] on a 13-inch iPad (Apple Inc., Cupertino, CA, USA). Although the recommended viewing distance for the MNREAD application is 40 cm, a fixed distance of 30 cm was used for all participants in the present study to maintain consistent testing conditions. This distance was selected because it was comparable to the mean preferred viewing distance previously reported in a low-vision cohort tested with the same application (30 cm, SD 10 cm) [10]. For participants who used near-vision spectacles, lensometry was performed to assess whether the habitual near correction was appropriate for the 30-cm testing distance. Habitual near corrections were considered appropriate when the near addition measured by lensometry was equal to or greater than the age-based near addition recommended for a 30-cm working distance. When the habitual addition was insufficient, or when participants did not wear near-vision spectacles, testing was performed with full distance refractive correction supplemented with an age-based near addition for a 30-cm viewing distance. Testing was performed in a separate examination room from that used for distance visual acuity testing. Ambient illuminance in the reading assessment room was measured with the same lux meter and was 220 lux, matching that of the visual acuity testing room.

**Fig 1:**
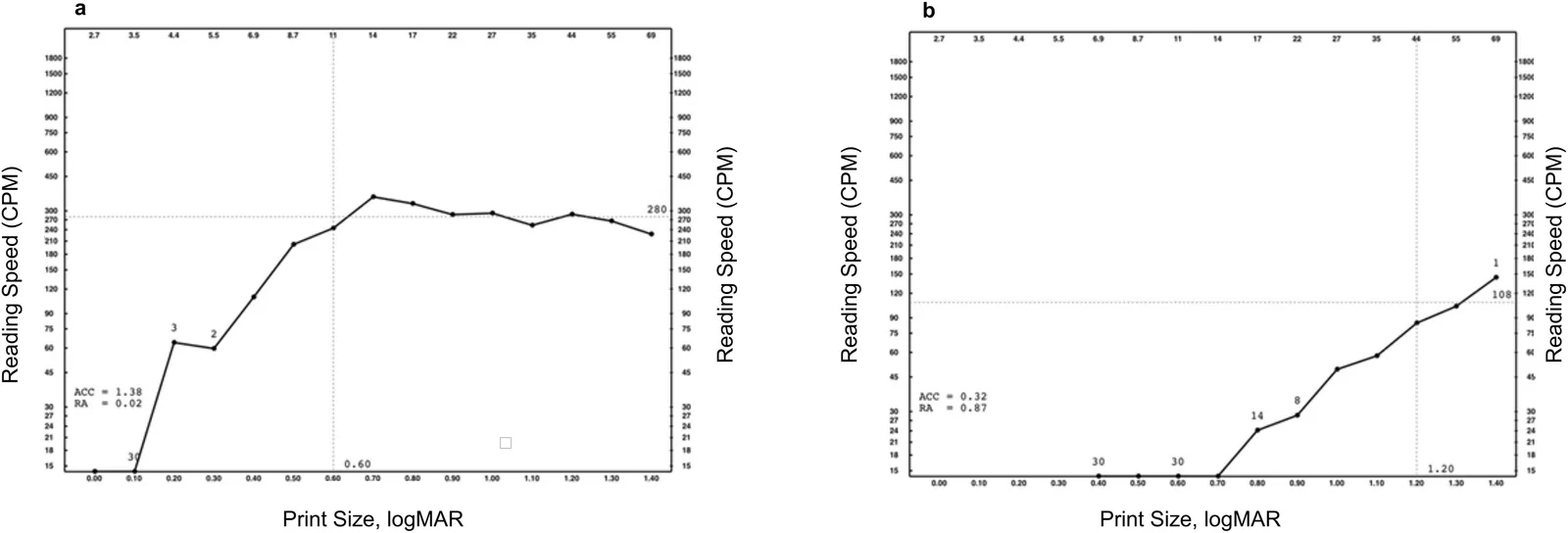
Typical and atypical MNREAD reading curves. (A) A typical reading curve, showing a smooth increase in reading speed as print size increases, followed by a plateau and gradual decline. (B) An atypical reading curve, lacking a clear plateau and showing marked fluctuations in reading speed (characters per minute [cpm]) across print sizes.

The application provides four parameters: maximum reading speed (MRS), critical print size (CPS), reading acuity (RA), and the reading accessibility index (ACC). CPS was defined as the smallest print size at which reading speed approached the maximum. These parameters have demonstrated acceptable inter-rater reliability in previous studies [14]. Reading curves were classified as typical or atypical based on the presence of a clear plateau in maximum reading speed (Fig 1).

### Statistical analysis

All analyses were performed using R version 4.5.2 (R Foundation for Statistical Computing, Vienna, Austria).

#### Primary analysis.

The prespecified primary analysis examined the association between the Functional Acuity Score (FAS) and critical print size (CPS), given the clinical relevance of CPS for estimating magnification needs. Associations between impairment measures (FVS, FAS, raw FFS, and adjusted FFS) and MNREAD parameters were assessed using Spearman rank correlation coefficients (rho).

#### Regression analysis.

Linear regression models were used to examine whether FAS was associated with reading outcomes after adjustment for demographic and clinical factors. Separate models were fitted for CPS and the reading accessibility index (ACC). Age, sex, and diagnosis category were included as covariates. For these analyses, diagnosis was dichotomized as glaucoma versus other causes of low vision, as glaucoma was the most common condition in this cohort. FAS was then added to the models to assess its independent association with each outcome. Model fit was evaluated based on the change in R^2^ after inclusion of FAS. Regression coefficients (B), standard errors (SE), t values, and two-sided P values are reported. Adjusted R^2^ values are presented to account for the number of predictors.

#### Model diagnostics.

Multicollinearity was assessed using variance inflation factors (VIF), with values <5 considered acceptable. Residual plots were inspected to check for major violations of model assumptions.

#### Secondary analyses.

FVS-related measures were compared between participants with typical and atypical reading curves using the Mann–Whitney U test. Because glaucoma was the most prevalent diagnosis, correlation analyses were repeated within this subgroup to assess the consistency of the main findings. Analyses other than the prespecified FAS–CPS association were considered exploratory. A two-sided P value <0.05 was considered statistically significant.

## Results

### Study population

**Fig 2:**
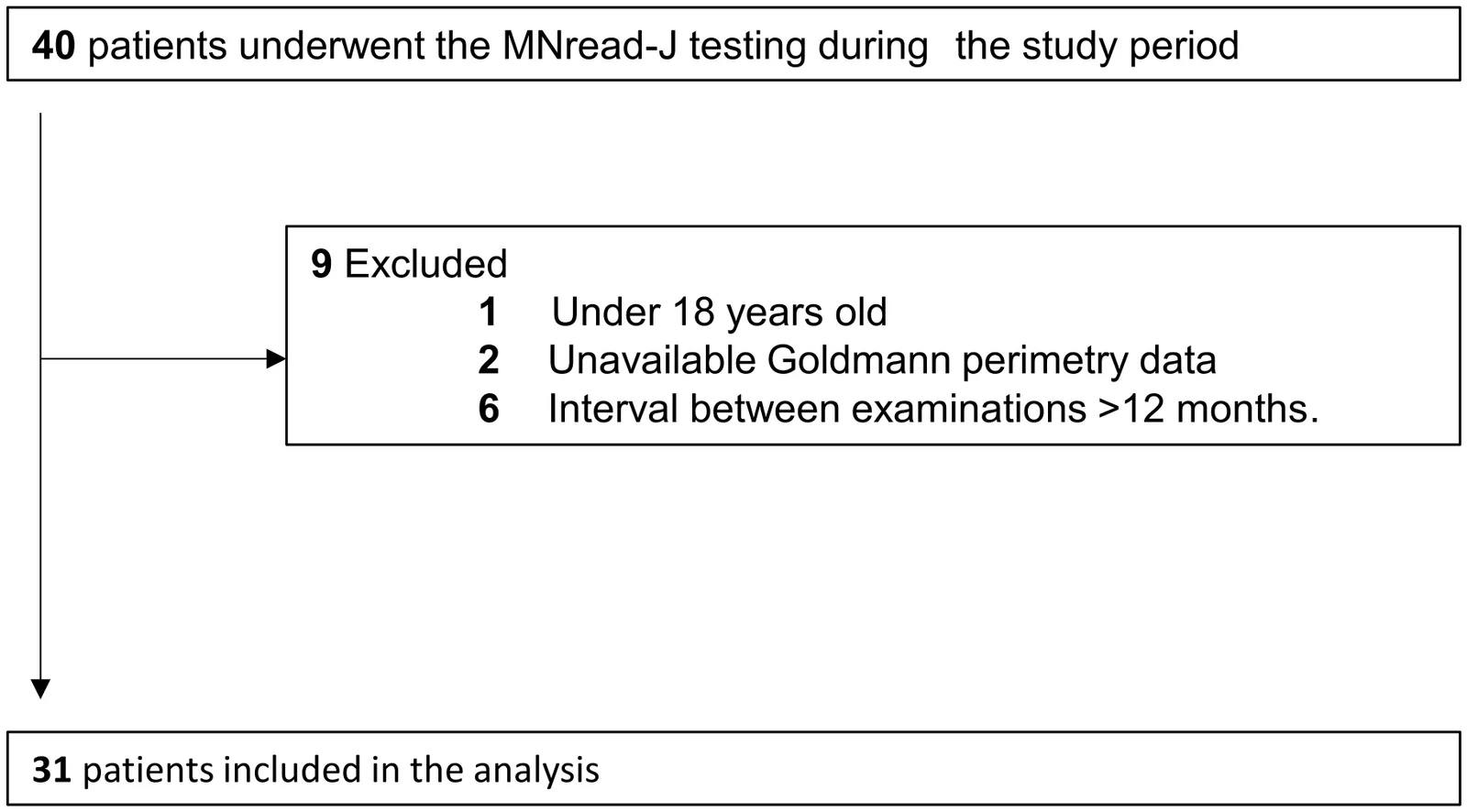
Flowchart of case inclusion and exclusion. The flowchart shows the participant selection process, including the number excluded and the reasons for exclusion.

Of the 40 patients screened, 31 met the inclusion criteria (Fig 2). Nine were excluded due to unavailable Goldmann perimetry data (n = 2), an interval exceeding 12 months between examinations (n = 6), or age < 18 years (n = 1).

Glaucoma was the most common diagnosis (18/31, 58.1%), followed by macular disease (4/31, 12.9%), optic neuropathy, and other conditions. Although an interval of up to 12 months was permitted, the interval between GP and MNREAD-J testing was short for most participants (median, 8 days; range, 0–78 days). Of the 31 participants, 15 underwent MNREAD-J testing using their habitual near correction, while 16 were tested using full distance refractive correction supplemented with an age-based near addition. Baseline characteristics are summarized in Table 1, and distributions of FVS-related measures and MNREAD-J parameters are shown in Table 2.

**Table 1:** Demographic and clinical characteristics of the patients (n = 31).

Characteristic	Value
Age, mean (SD), y	66.0 (14.5)
Sex, No. (%)	
Male	9 (29.0)
Female	22 (71.0)
Causative Disease, No. (%)	
Glaucoma	18 (58.1)
Macular disease	4 (12.9)
Retinitis pigmentosa	3 (9.7)
Optic nerve disease	3 (9.7)
Other retinal disease	3 (9.7)
Interval between tests, median (range), d	8 (0–78)
Correction type at MNREAD-J testing, No. (%)	
Habitual correction	15 (48.4)
Full correction	16 (51.6)

**Table 2:** Functional vision scores and MNREAD-J parameters.

Parameter	All Patients (n = 31), Mean (SD)	Glaucoma Patients (n = 18), Mean (SD)
MNREAD-J		
Typical reading curve, n (%)	14 (45%)	7 (39%)
MRS, cpm	150.1 (48.7)	158.3 (45.4)
CPS, logMAR	1.01 (0.49)	1.02 (0.55)
RA, logMAR	0.72 (0.44)	0.67 (0.48)
ACC	0.60 (0.22)	0.65 (0.22)
FVS-related Scores		
VAS-better eye	67.1 (22.3)	70.9 (17.6)
VAS-worse eye	36.7 (30.6)	29.2 (38.3)
FAS	61.1 (27.6)	62.1 (28.5)
FFS, with scotoma rule	56.6 (31.0)	66.3 (27.5)
FFS, without scotoma rule	43.1 (31.6)	46.6 (32.1)
FVS	34.0 (26.6)	36.6 (29.3)

Abbreviations: ACC, reading accessibility index; cpm, characters per minute; CPS, critical print size; VAS, Visual Acuity Scores; FAS, functional acuity score; FFS, functional field score; FVS, functional vision score; MRS, maximum reading speed; RA, reading acuity; SD, standard deviation

### Correlation analysis

#### FAS and reading performance.

**Fig 3:**
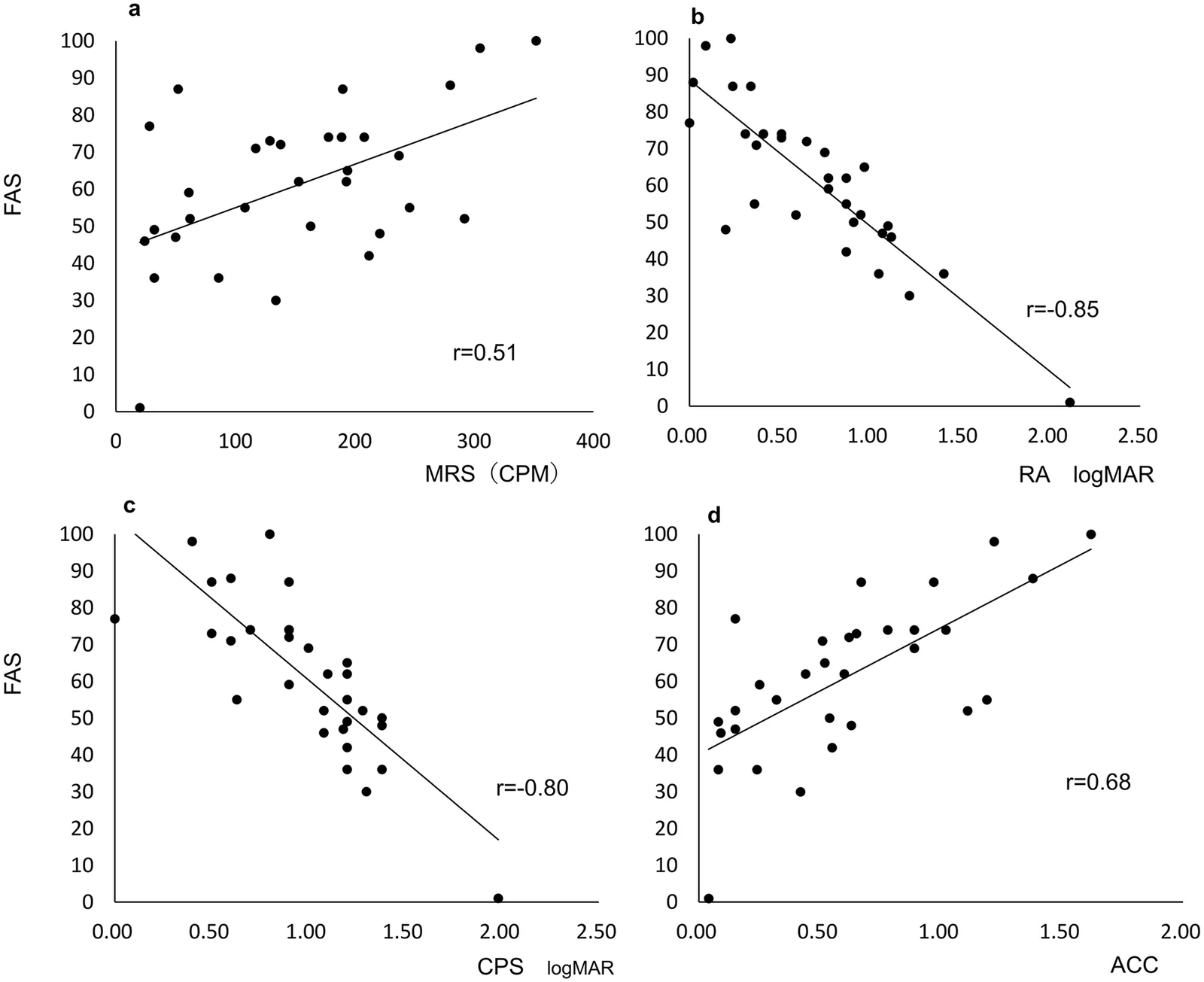
Correlations between the functional acuity score (FAS) and reading performance parameters. Scatter plots show the relationships between FAS and (A) maximum reading speed (MRS; characters per minute [cpm]), (B) reading acuity (RA), (C) critical print size (CPS), and (D) the reading accessibility index (ACC). Higher FAS values were associated with faster reading speed, smaller RA and CPS values, and higher ACC values. All correlations were statistically significant (all P ≤ 0.01). Spearman’s rank correlation coefficient (rho) is shown for each relationship.

FAS was highly correlated with better-eye VAS (rho = 0.963, P < 0.001), consistent with FAS functioning primarily as a weighted measure of monocular visual acuity. Among the impairment measures, FAS showed the strongest associations with MNREAD parameters, most notably CPS (rho = −0.82, P < 0.01) (Table 3, Fig 3).

**Table 3:** Spearman correlation coefficients (rho) between FVS components and MNREAD-J parameters.

Parameters	MRS		RA		CPS		ACC	
	rho	p	rho	p	rho	p	rho	p
All (n = 31)								
FVS	0.45	0.01	−0.40	0.03	−0.24	0.19	0.51	<0.01
FAS	0.43	0.01	−0.82	<0.01	−0.82	<0.01	0.69	<0.01
Raw FFS	0.49	<0.01	−0.31	0.09	0.03	0.88	0.45	0.01
Adjusted FFS	0.06	0.75	0.24	0.20	0.44	0.01	−0.04	0.85
Glaucoma(n = 18)								
FVS	0.45	0.06	−0.32	0.20	−0.20	0.44	0.41	0.10
FAS	0.53	0.02	−0.88	<0.01	−0.79	<0.01	0.71	<0.01
Raw FFS	0.52	0.03	−0.24	0.34	0.05	0.84	0.38	0.12
Adjusted FFS	−0.12	0.63	0.47	0.05	0.53	0.02	−0.29	0.24

Abbreviations: ACC, Reading Accessibility Index; CPS, critical print size; FAS, functional acuity score; FFS, functional field score; FVS, functional vision score; MRS, maximum reading speed; RA, reading acuity

Higher FAS values were associated with the ability to read smaller print and with greater reading accessibility. Similar associations were observed in the glaucoma subgroup (Table 3), suggesting that these findings were not driven solely by diagnostic composition.

#### Regression analysis.

Linear regression models were used to assess whether FAS remained associated with reading outcomes after adjustment for age, sex, and diagnosis (Table 4).

**Table 4:** Hierarchical multiple regression analyses of reading performance.

Model 1	Age	-0.010	0.005	-2.16	0.039	0.204	0.125		
	Sex	-0.208	0.135	-1.55	0.133				
	Diagnosis	-0.003	0.132	-0.02	0.984				
Model 2	Age	-0.006	0.003	-2.28	0.031	0.762	0.726	0.559	<0.001
	Sex	-0.087	0.077	-1.14	0.266				
	Diagnosis	-0.027	0.073	-0.36	0.719				
	FAS	-0.013	0.002	-7.82	<0.001				
**B Outcome: ACC**									
Model 1	Age	0.003	0.006	0.51	0.616	0.173	0.098		
	Sex	0.392	0.168	2.33	0.028				
	Diagnosis	0.136	0.165	0.82	0.418				
Model 2	Age	-0.001	0.004	-0.31	0.757	0.590	0.527	0.417	<0.001
	Sex	0.263	0.123	2.13	0.043				
	Diagnosis	0.161	0.118	1.36	0.185				
	FAS	0.013	0.003	5.14	<0.001				

Hierarchical regression models were used with demographic and diagnostic variables entered in Model 1 and FAS added in Model 2. Diagnosis was coded as glaucoma versus other causes of low vision. B values represent unstandardized regression coefficients. ΔR^2^ indicates the additional variance explained by FAS.

Addition of FAS improved model fit for both CPS and ACC, and FAS remained significantly associated with both outcomes in the fully adjusted models (Table 4). Age showed a modest independent association with CPS, and sex showed a modest independent association with ACC; diagnosis was not significantly associated with either outcome. Variance inflation factors were <1.4 for all predictors, indicating no evidence of problematic multicollinearity.

#### Associations of FVS and FFS with reading parameters.

FVS and raw FFS showed less consistent associations with reading outcomes than FAS, correlating with some but not all MNREAD parameters (Table 3). In the glaucoma subgroup, raw FFS showed no significant associations with MNREAD parameters. When the central scotoma rule was applied, adjusted FFS showed modest correlations with CPS in both the overall cohort and the glaucoma subgroup (Table 3), but was not associated with other MNREAD parameters.

#### Reading curve patterns.

**Fig 4:**
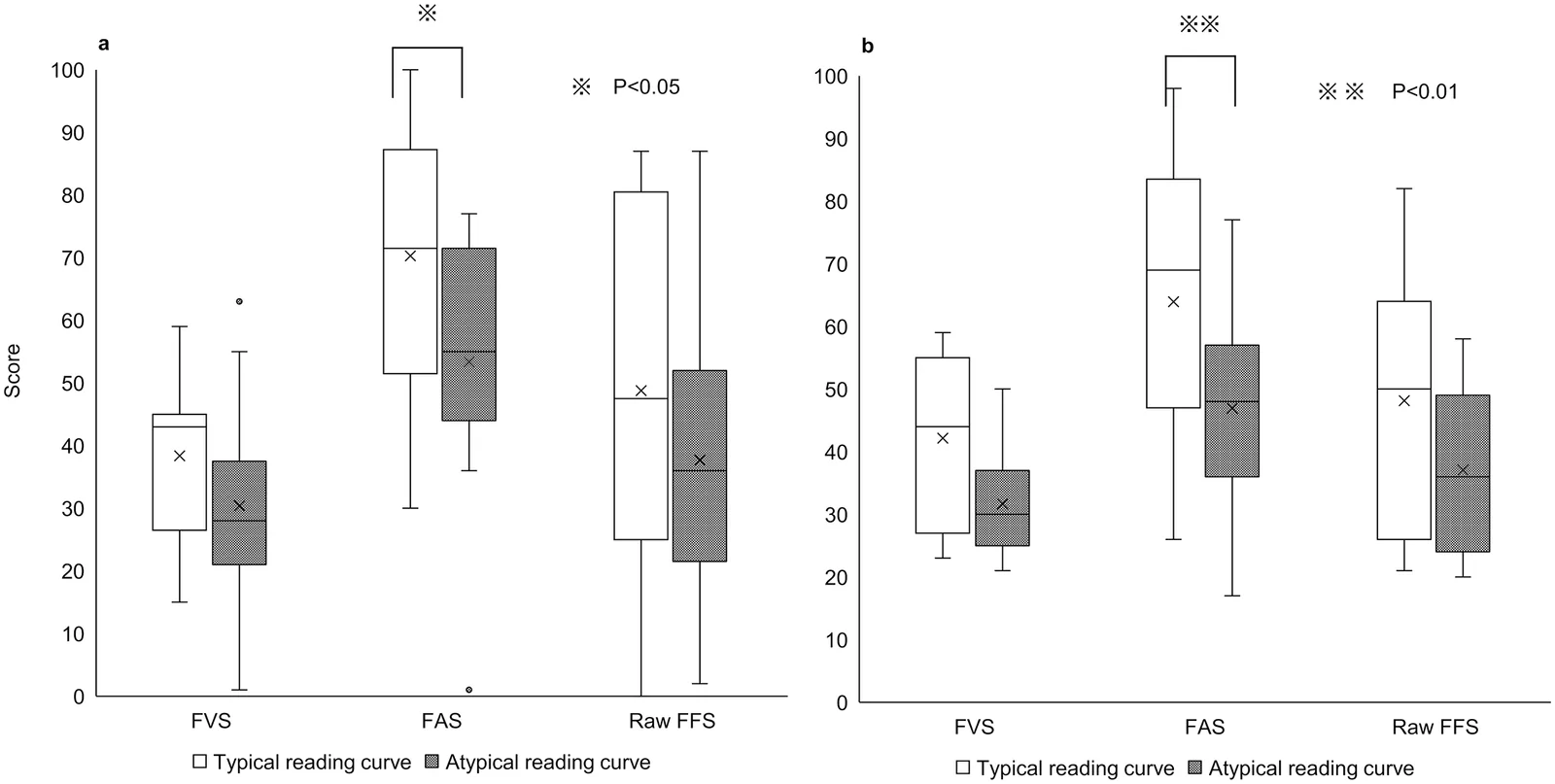
Impairment scores by MNREAD reading curve pattern. (A) All participants (n = 31). (B) Glaucoma subgroup (n = 18). FAS, FVS, and raw FFS were compared between participants with typical and atypical reading curves. FAS was significantly lower in the atypical group, whereas FVS and raw FFS did not differ significantly. Boxes represent the interquartile range, horizontal lines indicate the median, and whiskers indicate the range. Group comparisons were performed using the Mann–Whitney U test.

Seventeen of 31 participants (54.8%) showed atypical reading curves. Median FAS was lower in the atypical group than in the typical group (55.0 vs. 71.5; P = 0.03), with a similar pattern in the glaucoma subgroup (55.0 vs. 74.0; P < 0.01) (Fig 4).

## Discussion

This study examined whether the Functional Acuity Score (FAS), an impairment measure derived from routine visual acuity data, was associated with activity-level reading performance in individuals undergoing formal visual disability evaluation. Although the relationship between visual acuity and reading performance is well established, it is less clear whether standardized impairment measures used in disability evaluation correspond to activity-level outcomes such as reading, a distinction reflected in the ICF framework between body function and activity [1]. In this cohort, FAS showed the most consistent associations with reading parameters, whereas field-based measures showed weaker or less consistent associations.

### Functional acuity and reading performance

The close relationship between FAS and both CPS and ACC is consistent with the role of central spatial resolution in reading. Under standardized high-contrast conditions, reading speed is constrained by the number of letters that can be identified within a fixation—the visual span [5,15,16]. Resolution-dependent CPS represents the smallest print size at which reading speed approaches its maximum. Because optotype acuity and CPS both depend primarily on foveal and parafoveal resolution [5,15], their strong association is expected.

The FAS weighting scheme incorporates both monocular and binocular components because binocular vision represents typical daily viewing conditions. Previous studies have shown that better-eye and binocular visual acuity are similarly strong predictors of reading performance, with only small differences between them in many low-vision populations, in which binocular acuity often approximates that of the better eye [17]. In the present study, however, the difference in visual acuity between the two eyes was substantial (mean VAS, 67.1 ± 22.3 vs 36.7 ± 30.6). Because binocular summation decreases as interocular differences increase [18], performance in acuity-dependent tasks may have been largely determined by the better eye. Consistent with this interpretation, FAS was highly correlated with better-eye visual acuity (rho = 0.963), suggesting that, in this cohort, FAS largely reflected better-eye acuity despite its inclusion of both monocular and binocular components.

### Implications of substituting better-eye acuity for binocular acuity

Binocular visual acuity was not measured because it is not routinely assessed in Japanese clinical practice or visual disability evaluations. Better-eye VAS was therefore used in place of VAS_OU for all FAS calculations. Accordingly, in this cohort, FAS effectively functioned as a weighted score based on monocular acuity rather than a measure incorporating directly measured binocular acuity.

The direction and likely magnitude of the bias introduced by this substitution should also be considered. In individuals with normal or near-normal vision, binocular acuity is typically better than better-eye acuity by approximately 0.04–0.07 logMAR because of binocular summation. Using better-eye acuity in place of binocular acuity would therefore tend to underestimate binocular acuity and, consequently, FAS. In low-vision populations, however, particularly among patients with macular disease, binocular inhibition is not uncommon. In such cases, binocular acuity may be similar to or worse than better-eye acuity, so the substitution may introduce little systematic bias or may even overestimate true binocular acuity.

Rubin et al. showed in a large community-based sample that better-eye acuity alone can reasonably estimate binocular acuity and that, for acuity-dependent tasks such as reading, it predicts performance as well as binocular acuity [19]. In contrast, the AMA weighted algorithm has been reported to underestimate binocular acuity when the difference between the two eyes exceeds one line. This finding suggests that, although the substitution used in the present study was necessary for practical reasons, it may approximate binocular acuity more closely than the AMA formula in populations with marked interocular asymmetry.

The strong association between FAS and resolution-dependent reading performance may largely reflect the well-established relationship between better-eye visual acuity and reading performance, rather than an independent effect of the binocular weighting component of FAS.

In multivariable models adjusted for age, sex, and diagnosis, FAS remained associated with both CPS and ACC, and adding FAS increased model R^2^. This suggests that, in this cohort, a large part of the variation in resolution-dependent reading outcomes was related to central visual acuity. Previous studies have reported more modest associations. For example, Xiong et al. (2018) found that letter acuity does not fully explain variability in functional performance [20]. The relatively high R^2^ values observed here (up to 0.76) may reflect the wide range of disease severity in the sample and the characteristics of the MNREAD test, rather than information provided by FAS beyond that provided by standard letter acuity. The main advantage of FAS over simple better-eye visual acuity may therefore be its standardized administrative framework rather than additional predictive value.

Although FVS incorporates both acuity and visual field components, its association with CPS was weaker than that of FAS alone. This may reflect the task-specific nature of reading, which depends largely on central spatial resolution in the absence of large central scotomas. When acuity is the main limiting factor, adding a global visual field component may weaken the apparent association with reading outcomes.

### Conceptual basis and limitations of acuity-based functional scores

FAS is calculated from standardized letter acuity measurements, with weighting intended to reflect binocular viewing under typical daily conditions. This approach supports consistent and practical use in administrative settings but does not account for other aspects of visual function, such as contrast sensitivity, lighting conditions, or task-specific visual demands. Letter acuity also does not distinguish visual processing for recognition from that required for visually guided action [21].

### Field metrics and task specificity

Raw FFS showed little association with reading parameters. Goldmann perimetry reflects the extent of the global kinetic visual field, whereas reading depends mainly on central visual function [22,23]. Peripheral field loss is therefore unlikely to substantially affect reading unless central vision is also impaired [24]. The modest correlation between adjusted FFS and CPS may reflect application of the AMA “central scotoma rule,” which incorporates visual acuity into the field score [4]. Because the adjusted score includes an acuity component, some association with CPS would be expected. In contrast, field extent alone was not associated with resolution-dependent reading performance in this sample.

### Reading curve morphology

Participants with atypical reading curves had lower FAS values. Reduced central resolution may limit the stability of the MRS plateau. As acuity decreases, the effective visual span may narrow, and greater effort may be required to maintain stable fixation across different print sizes. In addition, estimation of reading parameters becomes less reliable at lower performance levels [22].

Other visual factors likely contribute to these findings. Fixation instability is known to affect reading speed, particularly in newly developed macular disease [25]. Macular pathology is also associated with increased parafoveal crowding [26,27]. In optic neuropathy and glaucoma, reduced contrast sensitivity may impair reading even when high-contrast visual acuity is relatively preserved [28,29]. These disease-specific mechanisms may influence the extent to which impairment scores correspond to functional outcomes, particularly under reduced contrast or non-ideal viewing conditions [30,31].

### Clinical and translational implications

From a practical standpoint, FAS may provide a useful reference for estimating likely reading limitations in settings where formal reading assessment is not available [9]. Because FAS is routinely calculated as part of disability evaluation procedures, it offers a readily accessible metric that requires no additional testing [4]. However, it does not capture factors such as crowding, contrast sensitivity, fixation stability, binocular interaction, or cognitive influences on reading [32].

In ICF terms, a body function measure such as FAS may approximate certain activity-level tasks that are strongly dependent on spatial resolution, but it does not encompass the broader context of functional vision [33].

Unlike Calabrèse et al. [10], who individually determined the preferred viewing distance for each low-vision participant, the present study used a fixed 30-cm viewing distance for all participants. Although this approach provided consistent testing conditions and was supported by the mean preferred viewing distance reported in that study, it may not have represented the optimal reading distance for every participant.

### Limitations

This study was retrospective and conducted at a single center. The sample size of 31 participants is a limitation. Although the primary FAS–CPS association was strong (rho = −0.82), the relatively small sample limits the precision of the estimates. The multivariable regression models also included several covariates relative to the number of participants, raising the possibility of overfitting. These models were therefore used mainly to examine whether the observed associations persisted after adjustment for covariates, rather than to develop a predictive model, and the results should be considered exploratory. In addition, participants were undergoing formal visual disability certification and may not represent the broader low-vision population.

Analyses other than the prespecified FAS–CPS association were exploratory. Nevertheless, the consistent patterns observed across related MNREAD parameters support the robustness of the primary finding. Binocular testing enhances ecological validity but may obscure interocular inhibitory effects [11,34]. Visual fields were measured using Goldmann perimetry rather than automated threshold perimetry or microperimetry, which may provide more detailed information on central sensitivity relevant to reading [35].

Sixteen of 31 participants (51.6%) were tested with full distance refractive correction plus an age-based near addition rather than their habitual near correction because lensometry indicated that the habitual correction was not appropriate for the 30-cm testing distance. This difference in near-correction strategy may have added variability to the MNREAD-J measurements. The cohort was predominantly non-macular, with glaucoma in 18 of 31 participants (58.1%) and glaucoma or other non-macular conditions in 27 of 31 (87.1%); only 4 participants (12.9%) had macular disease. Reading performance in macular disease may be affected by factors beyond central acuity, including fixation instability and parafoveal crowding [25–27]. Therefore, the association between FAS and reading performance observed in this study may not apply to patients with macular pathology. Studies including larger numbers of patients with macular disease are needed to determine whether these findings are applicable to this population. Adaptive strategies, such as eccentric viewing training [36] and low-vision aids [37,38], were also not systematically evaluated.

## Conclusion

In this predominantly non-macular cohort undergoing formal visual disability evaluation, FAS was strongly associated with resolution-dependent reading performance, an association that likely reflects the shared dependence of both measures on central visual acuity,　rather than an independent predictive role of FAS. Because FAS is routinely derived from visual acuity data, it may provide a practical estimate of resolution-dependent reading performance in similar populations when direct reading assessment is unavailable. In contrast, field-based scores were less informative for this task. Macular disease was underrepresented in the present cohort (4 of 31 participants); therefore, whether FAS is applicable to patients with macular pathology remains unclear and should be examined in disease-specific studies. Direct assessment of reading performance remains the reference standard in comprehensive low-vision care.

## Supporting information

S1 Data Anonymized dataset for the primary analyses. Minimal anonymized dataset required to reproduce the main correlation and regression analyses reported in this study.
(XLSX)
